# Gonad transcriptome analysis of pearl oyster *Pinctada margaritifera*: identification of potential sex differentiation and sex determining genes

**DOI:** 10.1186/1471-2164-15-491

**Published:** 2014-06-18

**Authors:** Vaihiti Teaniniuraitemoana, Arnaud Huvet, Peva Levy, Christophe Klopp, Emeline Lhuillier, Nabila Gaertner-Mazouni, Yannick Gueguen, Gilles Le Moullac

**Affiliations:** Ifremer, UMR 241 EIO, Labex CORAIL, BP 7004, 98719 Taravao, Tahiti, Polynésie Française; UMR 6539 LEMAR, Ifremer, BP 70, 29280 Plouzané, France; Sigenae, UR875, Auzeville, INRA, BP 52627, 31326 Castanet-Tolosan, France; GeT-PlaGe, Genotoul, INRA Auzeville, 31326 Castanet-Tolosan, France; UMR 241 EIO, Labex CORAIL, Université de la Polynésie Française, BP 6570, 98702 Faa’a, Tahiti, Polynésie Française; GeT-Purpan, GenoToul, UDEAR UMR 1065 CNRS/UPS/U1056 INSERM, CHU PURPAN, Place du Dr Baylac, TSA 40031, 31059 Toulouse Cedex 9, France

**Keywords:** *Pinctada margaritifera*, Gametogenesis, Transcriptome, Differential expression, Sex determinism

## Abstract

**Background:**

Black pearl farming is based on culture of the blacklip pearl oyster *Pinctada margaritifera* (Mollusca, lophotrochozoa), a protandrous hermaphrodite species. At first maturation, all individuals are males. The female sex appears progressively from two years old, which represents a limitation for broodstock conditioning for aquaculture production. In marine mollusks displaying hermaphroditic features, data on sexual determinism and differentiation, including the molecular sex determining cascade, are scarce. To increase genomic resources and identify the molecular mechanisms whereby gene expression may act in the sexual dimorphism of *P. margaritifera*, we performed gonad transcriptome analysis.

**Results:**

The gonad transcriptome of *P. margaritifera* was sequenced from several gonadic samples of males and females at different development stages, using a Next-Generation-Sequencing method and RNAseq technology. After Illumina sequencing, assembly and annotation, we obtained 70,147 contigs of which 62.2% shared homologies with existing protein sequences, and 9% showed functional annotation with Gene Ontology terms. Differential expression analysis identified 1,993 differentially expressed contigs between the different categories of gonads. Clustering methods of samples revealed that the sex explained most of the variation in gonad gene expression. K-means clustering of differentially expressed contigs showed 815 and 574 contigs were more expressed in male and female gonads, respectively. The analysis of these contigs revealed the presence of known specific genes coding for proteins involved in sex determinism and/or differentiation, such as *dmrt* and *fem-1 like* for males, or *foxl2* and *vitellogenin* for females. The specific gene expression profiles of *pmarg-fem1-like*, *pmarg-dmrt* and *pmarg-foxl2* in different reproductive stages (undetermined, sexual inversion and regression) suggest that these three genes are potentially involved in the sperm-oocyte switch in *P. margaritifera*.

**Conclusions:**

The study provides a new transcriptomic tool to study reproduction in hermaphroditic marine mollusks. It identifies sex differentiation and potential sex determining genes in *P. margaritifera*, a protandrous hermaphrodite species.

**Electronic supplementary material:**

The online version of this article (doi:10.1186/1471-2164-15-491) contains supplementary material, which is available to authorized users.

## Background

The blacklip pearl oyster *Pinctada margaritifera* (Linnaeus, 1758; Mollusca, Family Pteriidae) is a benthic bivalve occurring in the Indo-Pacific region, which is particularly abundant in the South Pacific, New Guinea, Hawaiian Islands, and Polynesia [1].

As marine bivalves are organisms of major economic interest, research has recently turned its attention to their genomics [2–4]. Black pearl farming is based on the culture of *P. margaritifera*, which a natural resource in French Polynesia. Research projects worked to develop ways to increase the pearl quality and to support sustainable development of pearl farming [5]. *P. margaritifera* is a biological model for biomineralization [6, 7] and reproduction [8, 9]; it is a sentinel species in the context of global change [5] and is also economically important for pearl production, a domain in which genetic selection can now contribute to quality improvement. It has been shown that the pearl aspect/quality is dependent on the genetics of the graft donor oyster [10]. Thus, the aim of the *P. margaritifera* selection program, is to breed families of graft donor oysters selected for their capacity to produce pearls of quality and/or particular colors and/or rapid growth [11]. The successful hatching of selected spat depends on the production of gametes and embryos from synchronous breeders raised in laboratory conditions. Controlled reproduction is therefore essential to set up a genetic improvement program. It relies on the knowledge of the underlying physiological mechanisms and factors controlling them [12–14].

Like other pearl oysters, including *P. mazatlanica* [15], *P. albino sugillata* [16], *P. imbricata* [17], *P. fucata* [18], and *P. radiata* [19], *P. margaritifera* is a protandrous hermaphrodite species [20] showing consecutive sexuality because individuals may change gender (from male to female) from the end of their second year onwards [13]. These observations would better correspond to sequential hermaphroditism. In all cases, simultaneous hermaphrodites and animals with undetermined status were uncommon. Sex ratio is the product of sex determination. The genetic and/or environmental process that establishes the gender of an organism [21], leads to specific molecular cascades transforming an undifferentiated gonad into a testis or an ovary [22]. In the animal kingdom, sex determination can be genetic (genetic sex determination, GSD), environmental (environmental sex determination, ESD), or the result of an interaction of both these factors [23]. In most bivalves, the main environmental factors affecting reproduction, and probably gender, are temperature, food availability [24, 25] and, to a lesser degree, photoperiod [26]. Environment stresses or farming processes were also reported to have important consequences for reproduction. Thielley (1993) [27] showed that gender changed when conditions were stressful, whether these were natural (temperature or food) or artificial (handling or cleaning).

Gender is determined by cascades of molecular signals that trigger differentiation of germinal cells into oocytes or spermatozoids. Since the discovery of *Sry* in mammals [28, 29], other master sex-determining genes have been characterized in vertebrates, such as *Dmy/dmrt1Yb*, *DM-W* and *DMRT1* in medaka (*Oryzias latipes*), *Xenopus laevis* and chicken, respectively [30–32], and, more recently, *amhy*, *Gsdf*, *Amhr2* and *SdY* in fishes [33–36]. Within these cascades, several genes were identified as playing key roles at a downstream level, including *foxl2* and *sox9* genes, crucial for ovarian and male differentiation, respectively [37, 38]. In invertebrates, particularly in marine mollusks displaying hermaphroditic features, data on sexual determinism, including molecular sex determining cascades, are rare. In the Pacific cupped oyster *Crassostrea gigas*, an alternative hermaphrodite mollusk, two genetic models of 2-genotypes and 3-genotypes have been proposed for sex determination. The first, proposed by Guo *et al.* (1998) [39], seems to be adequate and applicable to sex determination of *P. margaritifera*, suggesting a dominant male *M* allele and a protandric recessive *F* allele. *FM* oysters are true males (permanent males) and *FF* oysters are protandric males (males that can change into females) depending on other genetic or environmental factors. In the second model, Hedrick and Hedgecock [40] proposed 3 genotypes: *FF* for true female oysters, *MM* for true male oysters and *FM* for individuals that may mature as females or males. Whatever the model, the authors assumed that sex in *C. gigas* would be controlled by a single, and as yet unknown, major gene. Only a few downstream actors of the molecular cascade of sex determination/differentiation of this species have been identified, such as *Cg-DMI* for the male pathway, *Cg-foxl2* and its natural antisense transcript *Cg-foxl2os* for the female pathway [41–43].

In the genus *Pinctada*, no gene has yet been identified as an actor of the molecular cascade of sex determination. Recently, in the draft genome of *Pinctada fucata*, some gene models were identified as encoding reproduction-related genes possibly involved in germline differentiation (Pifuc-*vasa*-like, Pifuc-*nanos*-like) and sex determination (Pifuc-*Dmrt2*), but no evidence was found of their function [44].

In the present study, we analyzed the whole gonad transcriptome of the blacklip pearl oyster *P. margaritifera* using Illumina sequencing technology, and compared the means of expression patterns obtained in different sexes and stages of pearl oyster gonad. This study was designed i) to provide a better understanding of the molecular mechanisms underlying the reproductive cycle, and ii) to identify some genes of interest encoding proteins involved in sex determination and gonad development. These results are important resources for future research on reproduction in *P. margaritifera* and other marine hermaphrodite bivalves.

## Methods

### Animal material and tissue sampling

Five-year-old adult *P. margaritifera* (n = 150) from the Takaroa atoll (Tuamotu Archipelago, French Polynesia) were grown in the Vairao lagoon for two and a half years and brought to the Ifremer laboratory in Tahiti, French Polynesia, in groups of 20 between July and December 2011. The oyster gonads were immediately dissected. For each oyster, gonad tissues were sampled for RNA extraction and fixed for histology.

**Figure 1:**
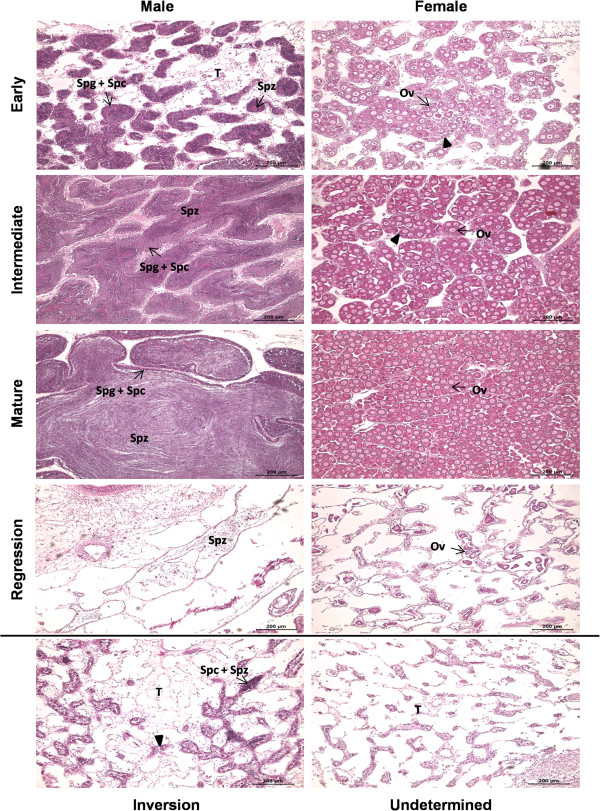
**Histological features of the ten categories of** ***P. margaritifera*** **gonad.** At the early stage, gonadic tubules, surrounded by abundant connective tissue, are less bulky and contain mostly spermatogonia and spermatocytes, and oogonia and oocytes in early development (►), in males and females, respectively. At the intermediate stage, tubules are large and the connective tissue is less abundant. In males, spermatogonia and spermatocytes form a ring at the periphery of tubules, while sperm occupy the central part. In females, oocytes in early development are still numerous (►) and those at the end of vitellogenesis begin to accumulate. At the mature stage, tubules are turgid, spermatozoids fill the entire lumen and spermatogonia and spermatocytes are reduced in number in males. In females, tubules contain only mature oocytes or those close to maturity. Regression stage is characterized by the retraction of tubules, which will then contain some residual spermatozoids in males, and some degenerating oocytes in females. Inversion category characterizes gonads in sexual inversion and thus presents both types of germ cells, male and female. And undetermined gonads are those which have no gametes and cannot therefore be associated with a sex. Ov: oocytes; Spc: spermatocytes; Spg: spermatogonia; Spz: Spermatozoid, and T: Connective tissue.

First, gonad development stage and sex were determined by histological methods and samples were classified into the ten different categories of gonadic tissues described in Figure 1: male and female at “Early” stage (the gonad is in early gametogenesis; Male: n = 17; Female: n = 14), “Intermediate” stage (the gonad is developing; Male: n = 58; Female: n = 30), at “Mature” stage (the oyster is ready to spawn; Male: n = 6; Female: n = 5), “Regressed” stage (the gonad has stopped generating gametes; Male: n = 9; Female: n = 6); “Inversion” (the gonad presents male and female gametes together; n = 2) and “Undetermined” (the gonad contains no gametes at all; n = 2). This classification was inspired by the reproductive scale proposed by Pouvreau *et al.* [8]. Secondly, in the resulting gonadic bank, 36 samples were selected for this study, according to their representativeness of a gonadic category, with four individuals per sex and stage except for the Inversion and Undetermined categories which were composed of two individuals.

For total RNA isolation and extraction, individual samples of gonadic tissues were conserved in RNAlater™ (Qiagen) (50 mg/mL) and stored at −80°C.

### RNA preparation

Total RNA was extracted using Extract-all (Eurobio) and treated by RTS DNAse™ (MO BIO Laboratories) following manufacturer’s instructions. RNA quality and integrity were checked by lab-on-chips analysis using the Agilent 2100 Bioanalyzer (Agilent Technologies). Because 28S and 18S rRNA fragments co-migrated in bivalves, we changed the threshold “Unexpected Ribosomal Ratio” pre-set at 0.7 to 1 in the software of the Agilent 2100 Bioanalyzer and also visually assessed the total RNA quality as demonstrated in Dheilly *et al.* (2011) [45]. RNA concentration was measured at 260 nm using an ND-100 spectrophotometer (Nanodrop Technologies). Samples were stored at −80°C until further use.

### cDNA library construction and Illumina sequencing

The cDNA libraries were made from the total RNA of the 36 individual samples, four individuals of each sex and stage, and two individuals of each undetermined and inversion categories. These RNA samples conformed to the required purity criteria (A260/A230 and A260/A280 > 1.8) and quality levels (RIN > 8) for cDNA library preparation for sequencing. The cDNA libraries were constructed using “Truseq RNA Sample Preparation v2” kits (Illumina), according to the manufacturer’s instructions. The mRNA molecules containing poly(A) were purified using magnetic poly(T) beads from 1–4 μg of each total RNA sample. A fragmentation buffer was added to break the mRNA into short fragments with an average length of 155 bp (120–210 bp). From these fragments, the first strands were synthesized using random hexamer primer and the second strands of cDNA were then synthesized. After purification and end repair, these short cDNA were ligated to the sequencing adapters (60 bp on each side) and enriched by polymerase chain reaction (PCR, 12 cycles). A range of final cDNA fragments of 320 ± 20 bp was selected using E-GEL SIZESELECT 2% (Invitrogen). The 36 cDNA libraries were normalized and grouped by six in six independent lanes, and finally paired-end sequenced on an Illumina HiSeq™ 2000 at the GeT-PlaGe core facility (Genome and Transcriptome – Plateforme Génomique, Toulouse, http://www.get.genotoul.fr), using TruSeq PE Cluster Kitv3 (2 × 100 bp) and TruSeq SBS Kit v3. All the obtained data were submitted to the Short Read Archive (SRA, http://www.ncbi.nlm.nih.gov/sra/) at the National Center for Biotechnology Information (NCBI), in Bioproject PRJNA229186 under the accession number SRP033217.

### Reference gonad transcriptome assembly and annotation

The dataset contains a large number of reads made from gonad tissue in different conditions. To limit the assembly problems encountered during the first tests, using all data at once, a two-step strategy was chosen. First, in order to build highly represented transcripts, 10 million random reads were assembled using ABySS with k-mer values of 35, 45, 55, 65 and 75. The resulting contigs were filtered according to their length, keeping those longer than 100 bp. The remainders were meta-assembled with MIRA. In order to keep only highly represented ones, the initial reads were remapped (BWA 0.6.1, [46]) on contigs and only the contigs with ≥ 500 aligned reads were kept. This produced 6,905 highly represented contigs, which were then used to filter corresponding reads in every sample read sets. The remaining reads were merged and assembled using the previously presented procedure. Finally, a contigs with over ten reads aligned in at least two samples or more than 50 aligned reads in a sample were filtered. To assess the assembly quality, all read sets were realigned on the contigs and had an alignment rate of at least 80%.

Assembled contigs were then functionally annotated in two ways. First, sequence similarities were sought by blastn/blastx with (i), a cut-off e-value of 10^−5^ against the following databases: *Pinctada fucata* Proteins (http://www.marinegenomics.oist.jp/ [47]), *Crassostrea gigas* Proteins (http://oysterdb.cn/ [48]), UniProtKB/Swiss-Prot Release 2012_06 of 13-Jun-2012, RefSeq Protein Index Blast of 27-Jun-2012, Pfam Release 26.0 of Nov-2011, RefSeq RNA Index Blast of 27-Jun-2012; and (ii), a cut off e-value of 10^−2^ against the following databases: TIGR Fugu FGI 3.0, TIGR ZebraFish ZGI 18.0, UniGene Fugu Build #9, UniGene Human Build #232, UniGene ZebraFish Build #125, Ensembl Ciona Transcripts CSAV2.0 67, Ensembl Fugu Transcripts FUGU4 67, Ensembl Human Transcripts GRCh37 67, Ensembl Tetraodon Transcripts TETRAODON8 67 and Ensembl ZebraFish Transcripts Zv9 67. Second, a Gene Ontology term (GO; http://www.geneontology.org/) was assigned if the best hits were already associated.

### Detecting Single-Nucleotide-Polymorphisms (SNPs) and Simple Sequence Repeats (SSRs)

The bam file produced for quantification were first filtered to eliminate poorly aligned sequences using an alignment quality threshold of 30 and a PCR duplicate filtering step (samtools v. 0.1.19-44428 cd). The read group information was added to each file by using picard tools (v. 1.88). To improve the variation calling, the alignment were reprocessed to re-align reads on medium size deletion spots and the base pair quality values of reads were recalibrated using GATK (v2.4-9-g532efad). All alignment files were jointly processed by GATK UnifiedGenotyper to produce the INDEL and the SNP formatted variant files (VCF). In addition, RepeatMasker v. open-3.3.0 (Smit and *al.*, unpublished data, http://www.repeatmasker.org/) was used to identify and localize simple sequence repeats (SSR or microsatellites) motifs. All type of SSRs from dinucleotides to hexanucleotides were searched using default settings (minimum total length = 20 bp).

### Sequences analysis

The amino acid sequences of invertebrates and vertebrates were aligned using ClustalW v2 software [49] and neighbor-joining trees with bootstrap values were constructed for phylogenetic analyses using the MEGA v6 software [50]. All the reference sequences for phylogenetic analyses were retrieved from GenBank and their corresponding accession number are listed in Additional file 1: Table S1.

### Differential expression (DE) analysis

Differential level expression of contigs between the ten different gonadic categories was tested using the DESeq package v1.11.3 [51] by use of the negative binomial distribution and a shrinkage estimator for the distribution’s variance. The analysis was performed after library normalization (function *estimatesizefactor*) of the contigs count table (RNAseq quantification, Additional file 2: Table S2), and following the standard procedure. Contigs considered as statistically significant differentially expressed were those showing an absolute value of Log2FoldChange > 2 i.e., exhibiting a fourfold increased expression under one of the conditions; and a *padj* < 0.001, p-value adjusted with a false-discovery rate (FDR) correction for multiple testing by Benjamini-Hochberg method [52].

A principal component analysis (PCA), using MeV v4.8.1 (MultiExperiment Viewer) [53] and a hierarchical clustering were applied to samples to cluster them according to the similarity of expression pattern of the statistically significant differentially expressed contigs. In addition, a K-mean clustering was performed, using R, on differentially expressed contigs, in order to cluster them based on similarity of expression between the different categories of pearl oyster gonads. The algorithm was set with ten centers (k = 10) corresponding to the number of gonadic categories.

### Real time PCR

In order to validate RNAseq data and expression profiles obtained from the DESeq analysis, real-time PCR was performed on 14 genes on the same samples. Among these genes, 11 were differentially expressed between the ten gonadic categories and distributed in different expression profiles; the remaining three genes did not show statistically differences in expression. Approximately 2.5 μg of total RNA of each sample obtained as previously described, were reverse-transcribed using M-MLV Reverse Transcriptase and amplified by real time PCR. The amplification reaction contained 5 μL 2X SYBR green qPCR Mix, 1 μL cDNA template, and 2 μL of each primer (1 μM) in a final volume of 10 μL. Each run included a positive cDNA control (reverse-transcribed pool of 0.5 μg total RNA of each sample) and a blank control (water) for each primer pair. Gene relative expression levels were calculated using 2 reference genes, *ef1a* and *gapdh1*, by the delta method [54], as follows: Relative expression_(target gene, sample x)_ = 2^^−^(Ct_target gene, sample x_ – Ct_reference gene, sample x_) = 2^-ΔCt^_(target gene, sample x)_. The relative stability of *ef1a* and *gapdh1* combination, considering the sex and reproductive stage of each gonad sample, was confirmed by using NormFinder [55]. A PCR efficiency (E) was estimated for each primer pair by determining the slopes of standard curves obtained from serial dilution analysis of the cDNA control to ensure that E ranged from 90 to 110%. The primers used for amplification are listed in Additional file 3: Table S3.

## Results

### Sequencing and assembly of the reference gonad transcriptome

To provide global view of the transcriptional changes between the ten pearl oyster gonadic categories, we assembled a reference gonad transcriptome *de novo*. To maximize the diversity of transcripts, the 36 cDNA libraries were sequenced and assembled together. Thus, Illumina sequencing generated 2,125,798,302 raw reads of 100 bp which after the assembly formed 70,147 contigs ranged from 100 to 17,424 bp with an average length of 1,294 bp (Table 1).

**Table 1:** **Summary statistics of** ***P. margaritifera*** **gonad transcriptome sequencing, assembly and annotation**

	Number	Percentage
**Reads**		
Total number	2125798302	
Number of base pairs	214705628502	
Length (bp)	100	
Average insertion size (bp)	198	
**Assembly**		
Number of contigs	70147	
Number of base pairs	90799729	
Number of GC base pairs	33298120	36.7%
Mapping rate of reads		91.8%
Average coverage of contigs (reads)	773.3	
Average coverage of contigs (rpkm)	9.8	
Average length of contigs (bp)	1294	
N50 of contigs (bp)	1948	
Longest contig (bp)	17424	
Shortest contig (bp)	100	
**Annotation**		
Unannotated contigs	26531	37.8%
Annotated contigs	43616	62.2%
Best hits (Hits):		
- *P. fucata* proteins	23522 (33448)	53.9% (76.7%)
- *C. gigas* proteins	9517 (23154)	21.8% (53.1%)
- Swissprot	362 (18736)	0.8% (43%)
- RefSeqProtein	2205 (22261)	5.1% (51%)
- RefSeq RNA	177 (5265)	0.4% (12.1%)
- Otherdatabases	7833 (19131)	18% (43.9%)

### SNP and SSR discovery

**Figure 2:**
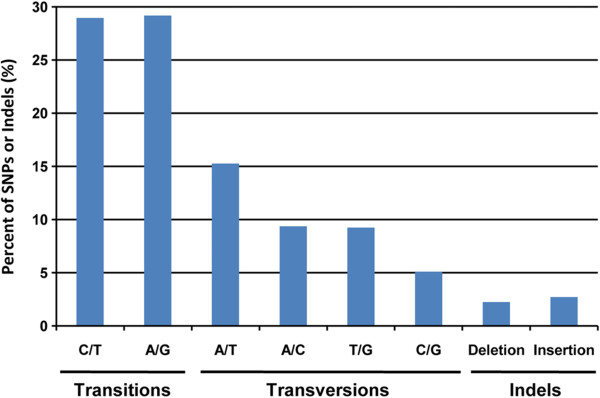
**Classification of single nucleotide polymorphisms (SNPs) including indels from the** ***P. margaritifera*** **gonad transcriptome.** Distribution (%) of each SNP and indel type. The overall frequency of all types of SNPs including indels was one per 23 bp.

Using GATK UnifiedGenotyper, we were able to identify 3,667,510 SNPs and 192,406 indels from 68,132 contigs. The overall frequency of all types of SNPs, including indels, was one per 23 bp (distributions presented in Figure 2). Transition occurred 1.5 times more frequently than transversion. A/T was the most abundant transversion (15.3%) and C/G (5.1%) the least abundant. Moreover, indels were less frequent than both transitions and transversions, with a frequency of one per 365 bp and a total proportion of around 5%.

In addition, using RepeatMasker, we identified 7,573 total SSRs from the gonad transcriptome of *P. margaritifera*, with a total number of 196 motifs (Table 2). Tetranucleotide repeats were the most frequent type, counting a total number of 4,259 (56.2%) with (CAGA)n as a major motif accounted for 33.5% of all tetranucleotide repeats. In the remainder, two groups with similar frequency were distinguished: one of di- and trinucleotides, which accounted for 30% of all SSRs, and one of penta- and hexanucleotide repeats, which accounted for less than 15% of all SSRs.

**Table 2:** **Summary of simple sequence repeat (SSR) types in** ***P. margaritifera*** **gonad transcriptome**

SSR type	Number of motifs	Count	%	Major motif	Count	%
Dinucleotides	6	1098	14.5	(GA)n	245	22.3
Trinucleotides	19	1151	15.2	(ATG)n	227	19.7
Tetranucleotides	52	4259	56.2	(CAGA)n	1426	33.5
Pentanucleotides	98	651	8.6	(TTTTG)n	69	10.6
Hexanucleotides	21	414	5.5	(CATATA)n	93	22.5
**Total**	**196**	**7573**				

### Functional annotation

**Figure 3:**
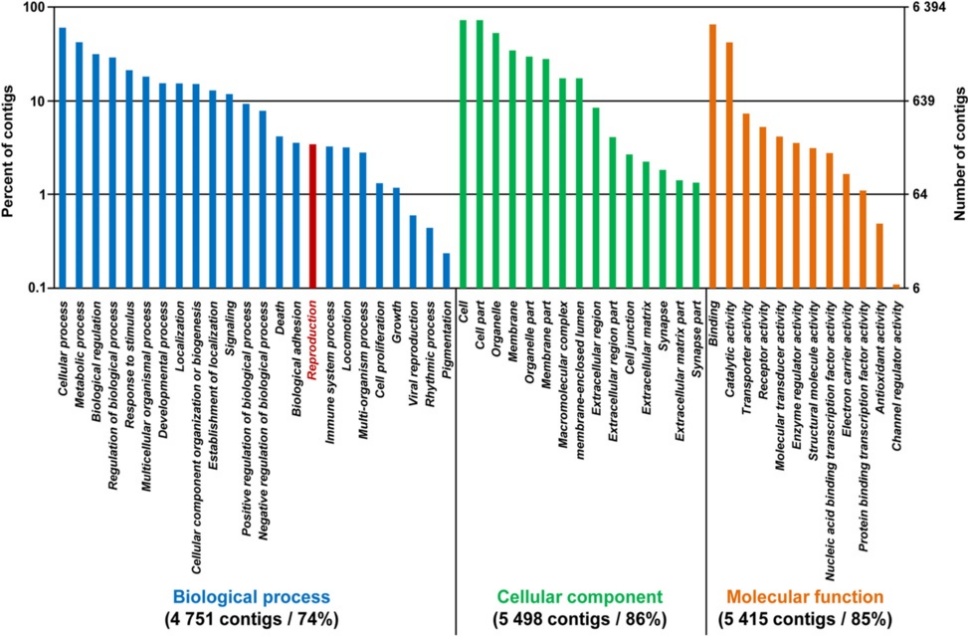
**Gene Ontology (GO) annotation of** ***P. margaritifera*** **contigs.** Distribution (%) of GO terms among the annotated contigs. GO ontologies are represented as general function categories. Among the 6,394 contigs with GO assignation, 4,751 (74%) are involved in biological process, 5,415 (85%) have a molecular function and 5,498 (86%) correspond to cellular component. In biological process, 220 (4.6%) contigs are implicated in reproduction.

Blastx/Blastn searches of the 70,147 contigs with the different databases revealed 43,616 (62.2%) with significant matches to existing protein sequences. Among these, 33,448 (53.9%) presented best hits with *Pinctada fucata* proteins, 9,517 (21.8%) with *Crassostrea gigas* proteins, and 2,744 with the three generic databases Swiss-Prot (362; 0.8%), RefSeq Protein (2,205; 5.1%) and RefSeq RNA (177; 0.4%) (Table 1).Gene ontology (GO) assignment was carried out on contigs in order to categorize the transcripts by putative function. Of 70,147 contigs, 6,394 (9%) were assigned with one or more GO term. Finally, 311,086 GO assignments were obtained, with a total of 10,001 GO terms. The assignments fall into the three major GO functional domains. Thus, among the 9%, 4,751 (74%) are involved in biological process, 5,498 (86%) are cellular components and 5,415 (85%) have molecular functions (Figure 3). Moreover, 4.6% (i.e., 220 contigs) were assigned to the biological process Reproduction.

### Genes related to sex determination/differentiation and gametogenesis

Of the 43,616 annotated contigs, a catalogue of 87 encoded for 67 putative proteins involved in sex determination/differentiation and gonad development (Additional file 4: Table S4). The functions of these genes are unknown in *P. margaritifera* but most of them had already been identified as playing an important role in these mechanisms in other organisms. For example, the relevant genes included *dmrt*, *sox9, fem-1* and *foxl2* as genes encoding proteins involved in sex determination/differentiation [38, 56–58], or genes encoding for proteins implicated in oogenesis and spermatogenesis as vitellogenin and testis-specific serine/threonine proteins kinase, respectively [59, 60].

#### Doublesex- and mab-3-related transcription factor

**Figure 4:**
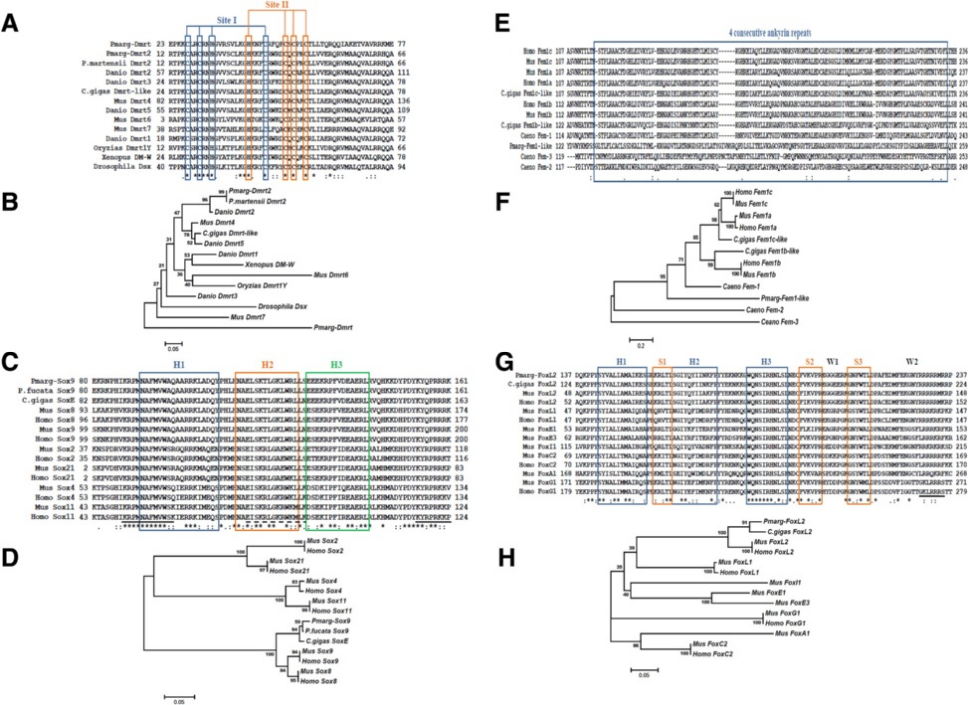
**Sequence analysis of four sex determination/differentiation-related genes in** ***P. margaritifera*** **. (A)** Amino acid sequence alignment of *P. margaritifera Dmrt* (Pmarg-Dmrt2, Pmarg-Dmrt) with the DM domain of various DMRT proteins. The zinc module consisting of intertwined CCHC and HCCC Zn^2+^-binding sites is shown with blue (Site I) and orange (Site II) boxes. **(B)** The phylogenetic tree generated using the DM domain of the DMRT proteins. **(C)** Amino acid sequence alignment of *P. margaritifera Sox9* (Pmarg-Sox9) with the HMG domain of various the SOX proteins. The three helices structuring the HMG domain are indicated with blue (H1), orange (H2) and green (H3) boxes. The nuclear export/localization signals (NES and NLS) are indicated in plain and dashed lines, respectively. **(D)** The phylogenetic tree generated using the HMG domain of SOX proteins. **(E)** Amino acid sequence alignment of *P. margaritifera fem1-like* (Pmarg-Fem1-like) with four consecutive ankyrin repeats (blue box) of various FEM proteins. **(F)** The phylogenetic tree generated using four consecutive ankyrin repeats of the FEM proteins. **(G)** Amino acid sequence alignment of *P. margaritifera foxl2* (Pmarg-Foxl2) with the Forkhead domains of various FOX proteins. The putative NLS sequence is underlined. The three helices (H1, H2 and H3; blue boxes), the two wings (W1, W2) and the β-strands (orange boxes) structuring the Forkhead domain are indicated. **(H)** The phylogenetic tree generated using the Forkhead domain of the FOX proteins. Alignments were generated using Clustal W2. Identical amino acids and amino acids with conserved similarities are indicated by asterisks and by dots/colons, respectively. The numbers of amino acid residues at the beginning and at the end of the different domains are indicated. The phylogenetic trees were generated using MEGA v6 via the neighbor-joining method. Numbers in the branches represent the bootstrap values (as a percentage) from 100 replicates. GenBank accession numbers of the reference sequences are listed in Additional file 1: Table S1.

Two transcripts encoding orthologs of the DM domain transcription factor were identified: a complete one of 993 bp (Contig_44478) with an open reading frame (ORF) of 825 bp, and a partial one of 1,073 bp (Contig_639) without an ATG codon but with a TGA stop codon at position 988 bp. The deduced amino acid sequences are 280aa and 329aa long, respectively, and contain the DM domain consensus sequence (from aa 12 to 66, Contig_44478; from aa 23 to 77, Contig_639) with conserved cysteines and histidines characteristic of the DMRT protein family (Figure 4A). From sequence comparison of conserved DM domains among various members of *Dmrt* family, our first *P. margaritifera* sequence was seen to share the highest amino acid identity with *P. martensii dmrt2* and zebrafish *Dmrt2* (100% and 95%, respectively), and the second with *P. martensii dmrt2* and mouse *Dmrt4* (59% and 58%, respectively). The sequence comparison reveals a conserved zinc module consisting of intertwined CCHC and HCCC Zn^2+^-binding sites [41]. The phylogenetic tree generated using the compared DM domains (Figure 4B) provided that the first *P. margaritifera dmrt* is grouped with *Dmrt2* with high bootstrap support (91) whereas the second is grouped with no family members; thus, we named them, *pmarg-dmrt2* and *pmarg-dmrt*, respectively.

#### SRY (sex determining region Y)-box

A complete transcript of a *Sox* ortholog of 2,327 bp (Contig_10720) shows an ORF of 1,392 (ATG: position 196 bp; TGA: position 1,585 bp). The deduced amino acid sequence is 463aa long and contains an HMG domain with two nuclear localization signals (NLS) and one nuclear export signal (NES) (Figure 4C). The amino acid sequence comparison of HMG domains from the *P. margaritifera* Sox and members of the SOX family revealed that *P. margaritifera sox* shared the highest identity rates with the *P. fucata Sox9* (99%), the Pacific oyster *SoxE* (*Cg-SoxE,* 98%), the human and the mouse *Sox8* and *Sox9* (93% and 91%, respectively). The phylogenetic tree generated using the compared HMG domains (Figure 4D) shows that *P. margaritifera sox* forms a group with *Cg-SoxE* and *P. fucata Sox9*,which is more closely related to the SoxE members (*Sox8* and *Sox9*) with high bootstrap support (100). *P. margaritifera sox* does not cluster with vertebrate *Sox8* or *Sox9*, but comparison of complete sequences shows, after *Cg-soxE* and *P. fucata Sox9*, maximal homology and identity (e-value: 2.10^−85^, identity: 51%) with the human *Sox9*. This transcript was named *pmarg-sox9*.

#### Sex determining protein fem

A complete sequence of 2,113 bp (Contig_1317) encoding a Fem ortholog was identified, showing an ORF of 912 bp (ATG: position 28 bp; TAG: position 937 bp). The deduced amino acid sequence is 303aa long and contains ankyrin repeats (four repeats, 122–259 aa) characteristic of the Fem proteins. Relationship between *P. margaritifera* Fem and other Fem proteins characterized in various organisms were investigated by sequence comparison of the four consecutive ankyrin repeats found in this ortholog (Figure 4E). *P. margaritifera* Fem shared highest identity with *C. elegans* Fem-1 (37%). The phylogenetic tree generated using the compared ankyrin repeats (Figure 4F) shows that *P. margaritifera* Fem protein forms an out-group on its own between Fem-2/Fem-3 and the Fem1 family members, to which it is strongly related (bootstrap support: 97). This *P. margaritifera* Fem mRNA was named *pmarg-fem1-like*.

#### Forkhead box L2

A complete *P. margaritifera* sequence of a *Forkhead box* ortholog of 1,624 bp (Contig_43072) showing an ORF of 1,134 bp (ATG: position 137 bp; TGA: position 1,269 bp) encodes an aa sequence of 377 bp containing the Forkhead domain consensus sequence (from aa 137 to 237), also known as “winged helix” domain, characteristic of the FOX protein family. The Forkhead domain protein sequence alignment of *P. margaritifera* Fox and members of the FOX family (Figure 4G) indicated that *P. margaritifera* Fox shares the highest aa identity rates with the Foxl2 of the Pacific oyster (95%), mouse and human (92%). The alignment also revealed that the Forkhead domain of Foxl2 proteins is conserved among species including the putative NLS sequences with basic amino acids at the C-terminal end (RRRRRMRR). The phylogenetic tree (Figure 4H) provided evidence that *P. margaritifera* Fox, named Pmarg-Foxl2, is grouped with other Foxl2 with high bootstrap support (100).

### Differential expression and cluster analysis

**Figure 5:**
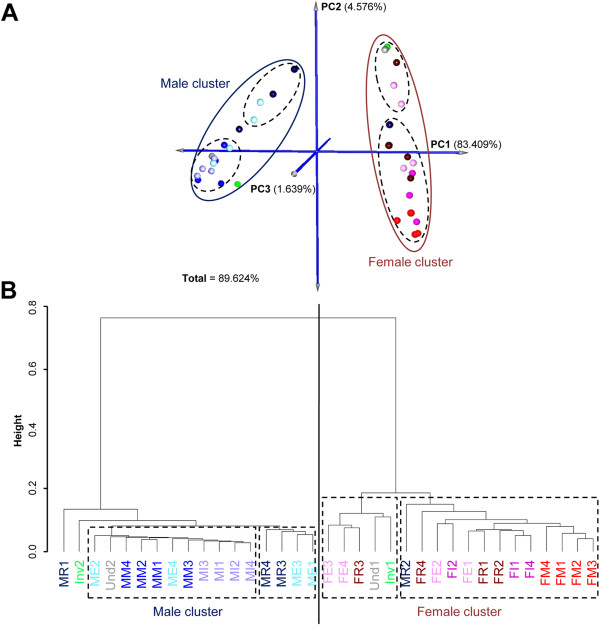
**Expression pattern analysis in gonad samples of** ***P. margaritifera*** **.** 3D Score plot using the first 3PCs identified by principal component analysis of all 1,993 contigs in the 36 individual oyster gonads **(A)**. Hierarchical clustering using Spearman’s correlation on all individual gonad samples **(B)**. Samples are divided into 2 main clusters based on their contig expression pattern, discriminating male and female gonads. Sky blue/pink or (M/F)E: male/female at early stage; cyan/magenta or (M/F)I: male/female at intermediate stage; blue/red or (M/F)M: male/female at mature stage; darkblue/darkpink or (M/F)R: male/female at stage of regression; green or Inv: gonads in sexual inversion; and grey or Und: gonad sex is undetermined.

**Figure 6:**
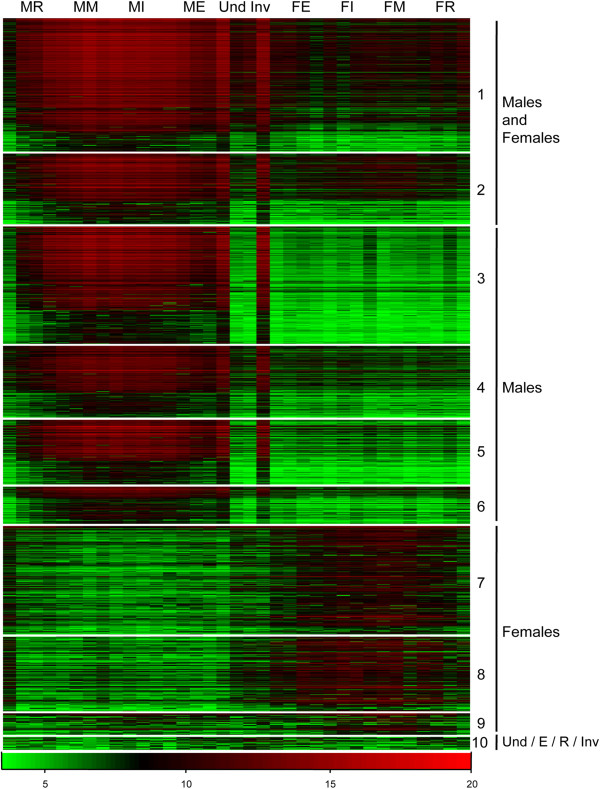
**Heat map of contigs differentially expressed between the ten different gonadic categories of** ***P. margaritifera*** **.** Clusters of the 1,993 differentially expressed contigs (rows) were obtained with K-means clustering using Spearman’s correlation. Contigs showing similar expression profiles on all samples (columns) clustered together. (M/F)E: male/female at early stage; (M/F)I: male/female at intermediate stage; (M/F)M: male/female at mature stage; (M/F)R: male/female at stage of regression; Inv: gonads in sexual inversion; and Und: gonad sex is undetermined. Four main expression profiles are identified: Males and Females (genes expressed in both sexes with expression changing over the course of gametogenesis, 561 contigs); Males (genes highly expressed in males with expression increasing over the course of spermatogenesis, 815 contigs); Females (genes highly expressed in females with expression increasing over the course of oogenesis, 574 contigs); and genes more expressed in undetermined, early, regressed gonads and in gonads in sexual inversion (43 contigs). Color represents the normalized expression after variance-stabilizing transformation (DESeq). Expression levels are depicted with a color scale, in which shades of red represent higher expression and shades of green represent lower expression.

In accordance with the absolute value of Log2FoldChange > 2 and *padj* < 0.001, the DESeq method identified 1,993 contigs differentially expressed between the ten gonadic categories (Additional file 5: Table S5). Among these contigs, 1,555 or 78% showed significant similarity to known proteins and 214 (10.7%) had a GO term.PCA and hierarchical clustering were applied on the 36 samples for the 1,993 contigs (Figure 5). Analysis of these two representations identified two main clusters: one female the other male. In the 3D score plot (Figure 5A), samples belonging to the same or to close gonadic categories appeared clustered together, whereas different samples, such as mature females (MF) and mature males (MM), appeared significantly further from each other. Sexes were discriminated by the first principal component (PC1), which explained 83.4% of the variation, with low component loading for males and high component loading for females. Stage of gonadic development seemed to be organized along the second principal component (PC2, 4.6%), with decreasing component loading from the early stage and regression stage to the mature stage. Focusing on PC2, two sub-clusters could be distinguished in each main cluster: one was represented by intermediate and mature stages whereas the other was represented by early and regressed stages. These sub-clusters were also found in the hierarchical clustering plot (Figure 5B). Then, a K-means clustering was performed on differentially expressed contigs and ten distinct clusters with similar expression patterns were produced (Figure 6). Biological interpretation of the data led us to group these clusters into four major gene expression profiles: i) contigs expressed in both male and female gonads, with an increasing expression over the course of gametogenesis (clusters 1 and 2); ii) contigs specifically expressed in male gonads, with an increasing expression over the course of spermatogenesis (clusters 3, 4, 5 and 6); iii) contigs expressed more in female gonads with an increasing expression over the course of oogenesis (cluster 7, 8 and 9); and iv) contigs more expressed in undetermined, early, regressed gonads and in gonads in sexual inversion (cluster 10).

Cluster 1 and 2 together included 561 contigs, of which 465 showed significant similarities to known proteins and 51 presented GO assignations. We found several genes known to be involved in male and female germ cell development in different organisms, and known to be implicated in varied processes such as chromatin condensation (*histone h4*, Contig_45897; and *histone h5*, Contig_62257), DNA replication and repair (*serine/threonie-protein kinase plk4*, Contig_4017; and *dna repair protein rad51 homolog 3*, Contig_2162); mitosis and meiosis with *cell division cycle proteins* (Contig_2034, Contig_4811, Contig_4931 and Contig_5497), *centromere proteins* (Contig_3993, Contig_49539), and *cyclins* (Contig_1166, Contig_2825, Contig_2971 and Contig_3583); transcription with several transcription factors (Contig_883, Contig_3574 and Contig_25707); post-transcriptional regulation and translation (*protein smaug homolog 1*, Contig_1040; and *apobec1 complementation factor*, Contig_5317); and apoptosis (*e3 ubiquitin-protein ligase zswim2*, Contig_1593; and *proteasome activator complex subunit 3*, Contig_2666). Moreover, because of a higher expression in male than in female, we also found in these two clusters of genes described as playing a role in spermatogenesis, such as *sperm-specific h1/protamine-like protein type 1* (Contig_6615), *testis-expressed sequence 11 protein* (Contig_1600) or *AMY-1-associating protein expressed in testis 1* (Contig_1857); and numerous genes involved in flagella/cilia and motility (*sperm flagellar proteins*, *sperm associated antigens*, *dyneins*, *kinesin-like proteins* or *coiled-coil domain-containing proteins*). In cluster 2, *pmarg-fem1-like* (Contig_1317) was also determined.

Among the 815 contigs with increasing expression over the course of spermatogenesis (clusters 3, 4, 5 and 6), 614 presented significant homologies with genes encoding known proteins and 72 were assigned with Gene Ontology. In these clusters, a high number (1.5 times) of contigs was found differentially expressed between males and females or were involved in different stages of spermatogenesis. Indeed, there were genes implicated in spermatocytogenesis, such as those encoding for synaptonemal complex proteins 2 and 3 (Contig_4991, Contig_730, Contig_1665 and Contig_3487); genes implicated in spermatidogenesis, including genes coding for proteins involved in spermatid differentiation and development such as meiotic recombination proteins (*r114l*, Contig_4686; *spo11*, Contig_6205; *dmc1*, Contig_4171; and *rec8*, Contig_6753) or testis-specific serine/threonine-protein kinase 1 (*tssk1*, Contig_3306, Contig_4127 and Contig_4767); and genes implicated in spermiogenesis, including genes encoding for the sperm motility kinase x (*smkx*, Contig_3725) or for the methyltransferase nsun 7 (*nsun7*, Contig_3944). For genes potentially involved in male sex determination, only *pmarg-dmrt* (Contig_639) was found in cluster 4. Finally, in these clusters, we identified genes coding for proteins implicated in fertilization, such as *adenylate cyclase type 10* (Contig_1091) or *protein hapless 2-b* (Contig_29401), and many genes known to play a role in the ubiquitin proteolytic system.

Among the 574 contigs with an increasing expression through the process of oogenesis (clusters 7, 8 and 9), 452 were annotated with known proteins and 89 were assigned in GO. We identified genes coding for proteins involved at different stage of oogenesis, such as oocytogenesis (*M-phase inducer phosphatase 1 and 3*, Contig_59221 and Contig_58023; *cyclin-dependent kinases regulatory subunit 1*, Contig_61458; *mitotic spindle assembly checkpoint protein MAD2A*, Contig_18326); and ootidogenesis, including proteins involved in ootide maturation (*proto-oncogene serine/threonine-protein kinase mos*, Contig_51755; *nanos-like protein 1*, Contig_43975). Many genes were also found to be implicated in glycoprotein biosynthesis and metabolism, including a female specific gene coding for vitellogenin-6 (*vit-6*, Contig_15150), and in lipid metabolism, e.g., fatty acid synthase or pancreatic lipase-related protein (*fasn*, Contig_22662, Contig_22663, Contig_41568, Contig_41569 and Contig_41569; *pnliprp1*, Contig_23348). In these clusters, we additionally determined the *pmarg-foxl2* (Contig_43072), *zglp1* (Contig_25360) and *ovo* (Contig_8141) genes encoding for proteins involved in female sex differentiation. Genes identified as playing a role in development were also found, such as *homeotic protein distal-less* (Contig_24570) and *frizzled-5* (Contig_36329). Moreover, a gene coding for a DNA (cytosine-5)-methyltransferase (Contig_674), known as playing a role in epigenetic mechanisms, was identified in the cluster 9 as differentially expressed in female mature gonads.

Forty-three contigs were found to be more expressed in undetermined, early, regressed gonads and in gonads in sexual inversion (cluster 10) and would decrease in expression along the gametogenic cycle. Among these contigs, 25 had significant similarity with known proteins and two contigs were assigned in GO. Some genes associated with the immune system were found, such as *toll-like receptor 1* (Contig_40001) and *complement C3* (Contig_35574).

### Highlights of expression patterns of putative sex determining genes

**Figure 7:**
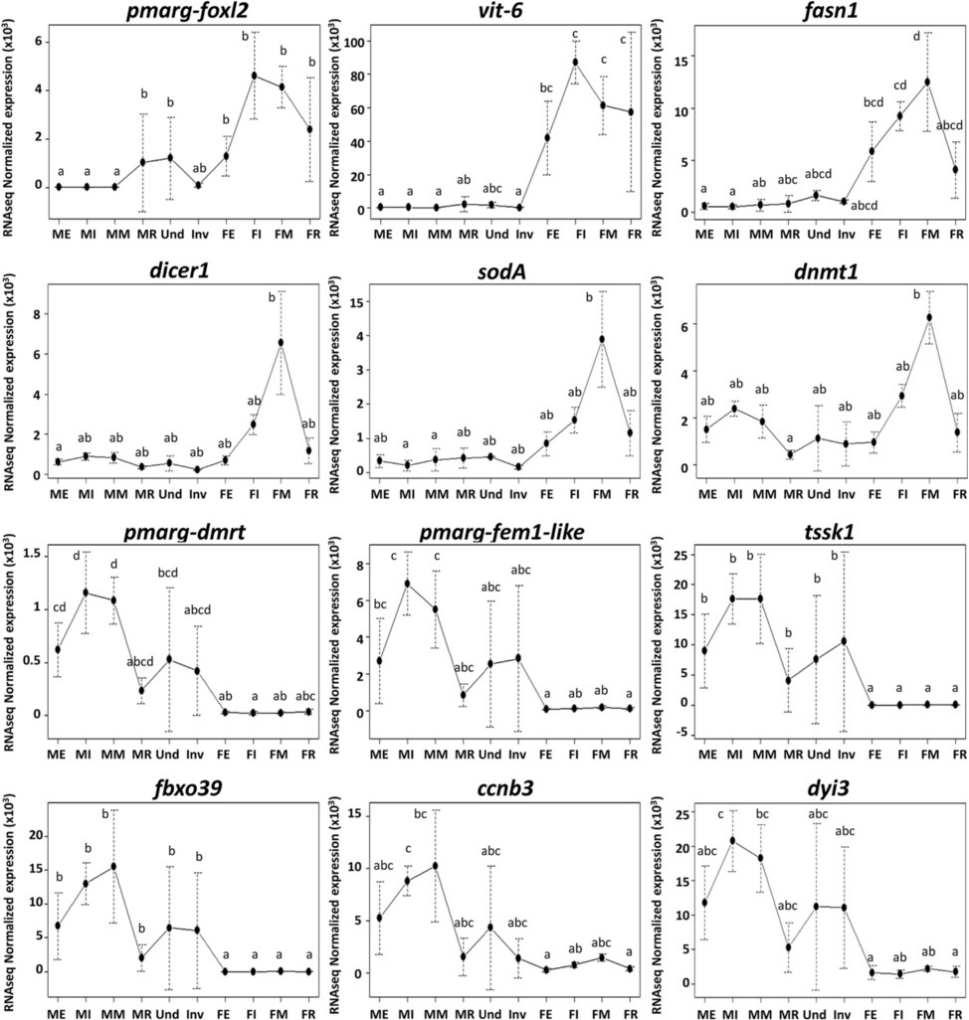
**Highlighted expression profiles of eight reproductive genes in** ***P. margaritifera*** **gonad samples.** RNAseq normalized expression (*estimatesizefactor*) profiles of some genes determined as differentially expressed over the ten different gonadic categories (sequenced samples). Vertical bars represent standard deviation for RNAseq data (n = 4 except for the undetermined and inversion categories where n = 2). Expression profiles are shown for specific genes expressed in female gonads: *forkhead box protein l2* (*pmarg-foxl2*, Contig_43072, cluster 7), *vitellogenin-6* (*vit-6*, Contig_15150, cluster 8), *fatty acid synthase* (*fasn*, Contig_22662, cluster 8), *endoribonuclease dicer* (*dicer1*, Contig_28823, cluster 9), *superoxide dismutase 1* (*soda*, Contig_25451, cluster 7) and *dna (cytosine-5)-methyltransferase 1* (*dnmt1*, Contig_674, cluster 9); three specific genes expressed in male gonads: *doublesex- and mab-3-related transcription factor-like* (*pmarg-dmrt*, Contig_639, cluster 4), *testis-specific serine/threonine-protein kinase 1* (*tssk1*, Contig_4767, cluster 5) and *f-box only protein 39* (*fbxo39*, Contig_3220, cluster 3); and three genes expressed in male and female gonads with a higher level in the male: *sex-determining protein fem-1-like* (*pmarg-fem1-like*, Contig_1317, cluster 2), *g2/mitotic-specific cyclin-b3* (*ccnb3*, Contig_1166, cluster 2) and *dynein intermediate chain 3, ciliary* (*dyi3*, Contig_3511, cluster 1). Development stage (E: early, I: intermediate, M: mature, R: regression) and sex (M: male, F: female, Und: undetermined sex, Inv: in sexual inversion) are indicated at the bottom of each figure. Different letters indicate statistically significant differences (DESeq, *padj* < 0.001).

The DE analysis identified some male and female sex determining/differentiation genes as differentially expressed between gonadic categories. The two potential male sex-determining genes found were *pmarg-fem1-like* (Contig_1317, cluster 2) and *pmarg-dmrt* (Contig_639, cluster 4). These genes showed a sexually dimorphic expression pattern in *P. margaritifera* with high expression in male gonad and a weak expression in female gonads passing through large variations of expression in the undetermined and inversion gonad categories (Figure 7). Moreover, the lowest mRNA level was observed in male regressed gonad, where it showed a similar level to the female gonads.

Furthermore, three genes identified as implicated in the female sex determination/differentiation pathway were found in female clusters: *pmarg-foxl2* coding for the Forkhead box protein L2 (Contig_43072, cluster 7), known to be essential for ovary differentiation and maintenance, and repression of the genetic program for somatic testis determination [61]; the GATA-type zinc finger protein 1 (*zglp1*, Contig_25360, cluster 8) and the protein ovo (*ovo*, Contig_8141, cluster 9), known to be involved in female germ line sex differentiation [62, 63]. These female genes presented an opposite expression profile compared to previously mentioned male genes, as shown in figure 7 for *pmarg-foxl2*. Here, high variations of expression were found in male gonads at regression stage and in gonads in which the sex was undetermined.

### RNAseq technical validation

**Figure 8:**
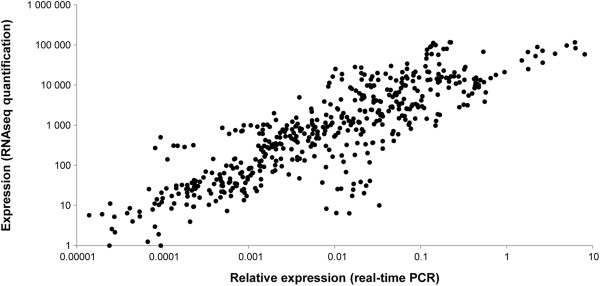
**Validation of the RNAseq quantification data using q-RT-PCR.** Fourteen genes were selected from differentially and non-differentially expressed contigs. There were 11 genes showing different expression levels depending on the sex and the maturity of the oysters: expression in both sexes (two genes from cluster 1: *tex11* [Contig_1600] and *dnah6* [Contig_2426]; and one gene from cluster 2: *ccnb3* [Contig_1166]), higher expression in males (one gene from cluster 4: *pmarg-dmrt* [Contig_639]; and two genes from cluster 5: *tssk1* [Contig_4767] and *mprb* [Contig_50490]), and higher expression in females (2 genes from cluster 7: *sox2* [Contig_37098] and *mos* [Contig_51755]; two genes from cluster 8: *vit-6* [Contig_15150] and *pnliprp1* [Contig_23348]; and one gene from cluster 9: *pbx4* [Contig_57857]). The three remaining genes, not differentially expressed but annotated as reproductive genes, were *pmarg-dmrt2* [Contig_44478], *dax1* [Contig_3226], and *vasa* [Contig_2898]. Their relative expression (to *ef1* and *gapdh1*) quantified by q-RT-PCR on samples used for sequencing were compared with those obtained using the RNAseq approach (normalized expression, *estimatesizefactor*) (R_S_= 0.86; *p* < 0.00001).

For technical validation of RNAseq data, real time PCR was performed on 14 chosen genes on the same individual samples previously used for the Illumina sequencing. Eleven genes were selected from the four main clusters described previously, plus three genes annotated as reproductive genes, and not identified as statistically differentially expressed. Between the normalized expression (*estimatesizefactor*) and the relative expression (relative to *ef1a* and *gapdh1*) of the RNAseq and the real time PCR data, there was a significant correlation (R_S_ = 0.86; *p* < 0.00001; Figure 8), which confirms the accuracy of the quantitative gene expression data.

## Discussion

RNAseq, based on recent next generation sequencing technologies, has been a widely used to obtain transcriptomic information on genes that are differentially expressed under contrasting biological conditions, including testis vs. ovary or different reproductive stages [64, 65]. This *de novo* technology is particularly suitable for non-model organisms for which genomic information is absent, weak or not relevant to the question addressed. In *P. margaritifera*, high-throughput expressed sequence tag (EST) pyrosequencing had already produced 276,738 sequences, but only from the calcifying mantle (in a study aimed at identifying shell matrix markers in the dynamic process of biologically-controlled biomineralization) [6]. Here, we proposed to unravel some molecular mechanisms involved in sex determination/differentiation and gametogenesis of an unusual alternative hermaphrodite invertebrate, the pearl oyster *Pinctada margaritifera* using Illumina-based RNAseq.

### Reference transcriptome of the gonad of *Pinctada margaritifera*

A reference gonad transcriptome was *de novo* assembled based on the sequencing of 36 cDNA libraries, each corresponding to gonad samples of different reproductive stages and sexes. Finally, 70,147 contigs with an average length of 1,294 bp were obtained. Approximately two-thirds of the contigs had significant matches against sequences of existing proteins and 9% were annotated using Gene Ontology terms. This result is higher than recently reported in transcriptomic analysis of other bivalves [66, 67], supposedly because the complete *C. gigas* and *P. fucata* protein databases have become available since their genomes were sequenced [47, 48]. Moreover, the higher number of contigs (10,294 more) with significant matches with *P. fucata* proteins than with *C. gigas* proteins would likely be due to the closer relationship between the two *Pinctada* species. From our sequencing effort, we identified a total of 196 *in silico* SSRs motifs and 3,667,510 putative *in silico* SNPs available for future genetics studies. This database and the available expressed sequences [6] will accelerate the development of both genomics and genetics in this commercially-important species and may also benefit the recently published draft genome of *Pinctada fucata*. For our main interest, this work successfully allowed the relation of numerous mRNA orthologs to sex determination/differentiation and gonad development genes.

### Identification of transcripts encoding proteins involved in sex determination and/or differentiation

The mechanisms governing sex determination and differentiation are highly variable among phyla. However while the genes at the top of hierarchy such as *Sry* in mammals or *sxl* in *Drosophila melanogaster* are not well conserved, the genes downstream in the sex determination pathway are more conserved.

The most conserved genes of this molecular pathway from invertebrates to human are *Dmrt*, a family of genes for *Doublesex* and *Mab-3*- related transcription factor [56]. The *Dmrt* genes encode a protein with an unusual zinc finger DNA-binding motif known as the DM domain [68]. At present, eight members of the family (*Dmrt1-8*) have been reported in vertebrates. In invertebrates, especially mollusks, orthologs of the DM domain transcription factor have been characterized from the oyster *C. gigas* (*Cg-DMl*) [41], the scallop *Chlamys farreri* (*Cf-dmrt4-like*) [69], and the pearl oysters *P. martensii* (*pmDmrt2* and *pmDmrt5*) [70, 71] and *P. fucata* (Pifuc-*Dmrt2* and Pifuc-*DM*-like A and B) [44]. Here, we characterized two *dmrt* orthologs in *P. margaritifera*: *pmarg-dmrt2* and *pmarg-dmrt*.

We also identified an ortholog of the *SoxE* gene in the pearl oyster: *pmarg-sox9*. In mammals, the transcription factor *Sox9* is the direct target of *Sry* and it is both necessary and sufficient for normal testicular development [72, 73]. It may induce the expression of another *SoxE* family member, *Sox8*, which participates in male gonadic differentiation and maintenance [74]. *Sox9* expression is highly up-regulated in developing male genital ridges [75–77]. The conservation of male-specific expression of *Sox9* suggests it is involved in normal sex determination in vertebrates. Recently, Santerre et al. (2014) [78] characterized the first *SoxE* ortholog (*Cg-SoxE*) in a Lophotrochozoa species, *C. gigas*. Expression pattern of *Cg-SoxE* suggested its involvement in early oyster gonadic differentiation, which includes sex determination.

A derived member of the little known family *Fem1* was characterized in *P. margaritifera*, *pmarg-fem1-like*. In the worm *C. elegans*, *fem-1* is a component of the signal transduction pathway controlling sex determination [58] and encodes FEM-1, an Ankyrin repeat protein. The *fem-1* gene, along with the *fem-2* and *fem-3* genes, are required for normal masculinization of somatic and germline tissue [79]. While it is thought that nematode sexual differentiation differs from that of vertebrates, a *Fem1* gene family, encoding proteins highly related to *C. elegans* FEM-1, was described in mouse and in human, consisting of three family members, *Fem1a*, *Fem1b* and *Fem1c* [80–83].

*Forkhead box l2* (*Foxl2*), which encodes a protein belonging to the Forkhead/winged helix family transcription factors, is one of the most conserved genes involved in the differentiation and maintenance of the ovary in vertebrates. In invertebrates, orthologs of *Foxl2* have been characterized, but without a good understanding of their role in reproduction [84–87]. In mollusks, orthologs of *Foxl2* have been also identified, such as in *C. gigas Cg-foxl2* [42] and the scallop *Chlamys farreri Cf-foxl2* [88], showing a sexually dimorphic pattern, in favor of female. In the present study, we identified a *foxl2* ortholog in *P. margaritifera*, *pmarg-foxl2*, which presents a highly conserved Forkhead domain characteristic of the FOX protein family.

This study therefore helped us to identify orthologs of genes encoding proteins known as sex determination/differentiation actors in other organisms. Their function in *P. margaritifera* remains to be examined. Functional approaches such RNAi technology, now available in marine bivalves [89], would help in deciphering the role of these genes in pearl oyster. Nevertheless, the gene expression pattern obtained by our RNAseq approach is a first step toward understanding their role in *P. margaritifera.*

### Sex is the main driver of gonad gene expression

Principal Component Analysis (PCA) revealed that the main variation in gene expression corresponds specifically to the sexes and gonad developmental stages. The sex factor alone explained more than two thirds of the variance in gonad gene expression. This strong sex effect was also revealed by gene expression profiling of the gonads of other marine bivalves with different reproductive physiology: the Pacific cupped oyster *Crassostrea gigas*, an alternative and irregular protandrous hermaphrodite marine bivalve [90]; the Pacific lion-paw scallop *Nodipecten subnodosus*, a functional hermaphrodite [65]; and the European clam *Ruditapes decussatus*, a gonochoric mollusk species that reproduces annually and shows sexual dimorphism [91]. In each case, molecular mechanisms specific to sex were so important that whatever the sexual determinism, the main variations in gene expression were between male and female rather than through gametogenesis development. Sex determination occurs at the onset of this distinction and is a crucial question in marine bivalves, especially in the pearl oyster, which has important environmental and aquaculture roles. From the mRNAs characterized with best hits of mRNA encoding major genes of sex determination in other organisms, few appeared differentially expressed, and positioned in clusters. The EST annotated as the female-specific gene *foxl2* showed a sexually dimorphic expression toward females and appeared differentially expressed over the course of vitellogenesis, with a significantly higher increase of expression between the “early” and “intermediate” stage females, possibly in agreement with a specific ovarian differentiation role. In the Pacific oyster, *Cg-foxl2* was demonstrated to indirectly participate in male gonadic differentiation, based on down-regulation induced by *Cg-foxl2os*, its natural antisense transcript [42, 43]. No information is currently available on the existence of a putative natural antisense transcript of *pmarg-foxl2* in *P. margaritifera*; this could be explored in combination with the role of *pmarg-foxl2*. Concerning male sex determining genes, only *pmarg-fem1-like* and *pmarg-dmrt* appeared significantly differentially expressed between conditions: over-expressed in *P. margaritifera* males and with an increasing expression over the course of spermatogenesis. In *C. gigas*, the kinetic of DMRT-like mRNA expression suggested that it plays a role in the development of the gonad (i.e., proliferation of spermatogonia and differentiation of Sertoli cells as found for *dmrt1* in vertebrates), though without any evidence of a sex-determinism function [41]. *fem-1* is a sex-determining gene that is required in *Caenorhabditis elegans* for the development of the male body and for spermatogenesis in males and hermaphrodites [58]. When males or hermaphrodites are *fem-1*(−), due to null mutations, animals develop as females. Here, the significant mRNA level of these two genes observed through the spermatogenesis of *P. margaritifera* might also maintain male development and spermatogenesis going, whereas repression of these two genes mRNA might provoke a sperm-oocyte switch, as observed in chicken for *dmrt1* [32] and in *C. elegans* for *fem-1* [92]. Only a few pearl oysters in undetermined or inversion stages were examined by histology, but high variance of their mRNA level was observed distinguishing two opposite patterns, one similar to repression and one like steady state levels of *pmarg-dmrt* and *pmarg-fem1-like* mRNA over the whole course of spermatogenesis. Non-destructive gonad biopsy allowing gene expression analysis in animals showing opposite patterns at undetermined or inversion stages may explain whether *pmarg-dmrt* and *pmarg-fem1-like* or *pmarg-foxl2* would induce sex inversion. *pmarg-dmrt* and *pmarg-fem1-like* seem to be repressed in male gonads at regression stage, as are many other specific male genes, whereas *pmarg-foxl2* seems to be more highly expressed at this stage. These results suggest that, physiologically, this stage of male regression is close to the female state. Indeed, undifferentiated, regression and inversion stages constitute the putative sex-determining time window in *P. margaritifera*, although the latter is potentially too late. Similarly, in *C. gigas*, a successive but not systematic protandric hermaphrodite species, the sex-determining time window may occur around the end of a reproductive cycle and the beginning of the next [93].

### Spermatogenesis

In male gonads, all male specific genes revealed an increasing expression over the course of spermatogenesis from early development to mature stages. No decreasing mRNA variation was observed. This result may highlight the asynchronous and continuous nature of the reproduction of *P. margaritifera*. Among the 815 contigs more expressed in males, several were identified as corresponding to genes involved in the ubiquitin proteolytic system (E3 ubiquitin-protein ligases, Kelch-like protein, F-box only proteins and ubiquitin carboxyl-terminal hydrolases), which have been shown to affect the sperm proteasomes in mice because a defect in these genes has negative effects on sperm efficiency [94]. The successful fertilization affected by sperm proteasomes relies on sperm capacitation. The ubiquitination-proteasome system has different levels of implication in fertilization: elimination of defective spermatozoa [95] and degradation of the proteinaceous vitellin egg coat [96]. Thus, testing these specific genes in relation to sperm quality in *P. margaritifera* would be of great interest, both from aquaculture and environmental perspectives.

### Oogenesis

We identified 574 genes potentially involved in oogenesis. Most of these genes appeared to have an increasing expression over the oocyte maturation process. Genes involved in the metabolism of glycoproteins and lipids, major components of the yolk envelop and yolk reserves, such as *vit-6*, *fasn* and *pnliprp1*, can be considered good targets to illustrate this process. Furthermore, maturation of oocytes includes the storage of mRNA, maternally transmitted to the embryo before the start of embryonic transcription [97]. Several genes found highly expressed in mature and known to be involved in embryo development might represent some of these maternal mRNAs. The high expression of the *cpeb1-b* gene coding for the cytoplasmic polyadenylation element binding protein 1-b, and the high expression of the *dicer1* gene coding for the endoribonuclease DICER may affect the translation or destruction of these mRNA through polyadenylation [98] or RNA interference mechanisms [99] during oocyte maturation. In addition, it has been shown in some vertebrates, that the oocyte quality is linked to the high expression of genes involved in antioxidant defense. Indeed, due to their aptitude to neutralize reactive oxygen species, they promote the lifespan of the embryo during its development [100]. Here, we found in mature stage, high expression of *sodA* gene coding for the superoxide dismutase-A which can be interpreted as similar process in *P. margaritifera*, and which may indicate a maternal investment in protection of the offspring which have to adapt to a fluctuating environment, especially at the beginning of their development.

Finally, DNA methylation of pearl oyster gametes is a potentially relevant direction for future studies. It is now known that epigenetic transitions can be important at defined stages of gametogenesis and during meiosis of germ cells [101, 102]. Methylation is faithfully recapitulated by the action of the maintenance methyltransferase *Dnmt1* which appeared here to be significantly modulated at the mRNA level during *P. margaritifera* gametogenesis so as to be up-regulated at the female mature stage. To date, *C.gigas* is the only such marine environmentally sensitive species to have been extensively studied in the epigenetic domain [103–105].

## Conclusion

The most significant outcome of our study is the identification of transcripts that improve our understanding of the specific reproduction of the marine bivalve *P. margaritifera* and enable us to produce lists of relevant candidate genes for further studies aimed at controlling reproduction of this species and thus supporting the sustainable development of pearl farming in French Polynesia. Among the candidate genes that appeared differentially expressed over the course of the gametogenesis or between sexes, *pmarg-dmrt*, *pmarg-fem1-like* and *pmarg-foxl2* would make good starting points for further functional research on sex determinism of the pearl oyster *P. margaritifera*. More specific and precise individual investigation is now needed to elucidate their role, using functional studies such as RNA interference.

Furthermore, a meta-analysis of the transcriptome of the gonad of several marine bivalves displaying different reproductive physiology, such as the alternative and irregular protandrous hermaphrodite oyster *Crassostrea gigas* [90], the functional hermaphrodite lion-paw scallop *Nodipecten subnodosus* [65], and the gonochoric European clam *Ruditapes decussatus* [91], would be interesting to carry out in order to shed light on the complex molecular cascade of sex determinism in marine mollusks.

## Availability of supporting data

Transcriptome data supporting the results of this article are available in the NCBI Short Read Archive (SRA, http://www.ncbi.nlm.nih.gov/sra/) in the Bioproject PRJNA229186 under accession number SRP033217.

## Electronic supplementary material

Additional file 1: Table S1: Genbank accession numbers of the reference sequences used for sequence analysis. (XLSX 10 KB)

Additional file 2: Table S2: RNAseq quantification: contig counts. (XLSX 18 MB)

Additional file 3: Table S3: Primer sequences. (XLSX 17 KB)

Additional file 4: Table S4: *P. margaritifera* genes potentially involved in sex determination/differentiation and gametogenesis. (XLSX 19 KB)

Additional file 5: Table S5: Differentially expressed contigs and their best annotations. (XLSX 595 KB)

## Abbreviations

GO: Gene ontology
PCR: Polymerase Chain Reaction
SNP: Single Nucleotide Polymorphism
SSR: Simple Sequence Repeat
DE: Differential Expression
PCA: Principal Component Analysis
E: PCR Efficiency
ORF: Open Reading Frame
EST: Expressed Sequence Tag.
